# Complete Genome Sequence of Bovine Polyomavirus Type 1 from Aborted Cattle, Isolated in Belgium in 2014

**DOI:** 10.1128/genomeA.01646-15

**Published:** 2016-03-03

**Authors:** Steven Van Borm, Toon Rosseel, Isabelle Behaeghel, Marc Saulmont, Laurent Delooz, Thierry Petitjean, Elisabeth Mathijs, Frank Vandenbussche

**Affiliations:** a Molecular Platform, Veterinary and Agrochemical Research Center, Ukkel, Belgium; b Interactions and Surveillance, Veterinary and Agrochemical Research Center, Ukkel, Belgium; c Regional Association for Animal Registration and Health (ARSIA) asbl, Ciney, Belgium

## Abstract

The complete and fully annotated genome sequence of a bovine polyomavirus type 1 (BPyV/BEL/1/2014) from aborted cattle was assembled from a metagenomics data set. The 4,697-bp circular dsDNA genome contains 6 protein-coding genes. Bovine polyomavirus is unlikely to be causally related to the abortion cases.

## GENOME ANNOUNCEMENT

From July until November 2014, an increase in the number of bovine abortions showing atypical icteric appearance and splenomegaly was observed in Belgium (1). In addition to specific diagnostic efforts to diagnose pathogens known to be associated with bovine abortion, a viral metagenomics workflow was applied to samples (liver, spleen, placenta, abomasal fluid) from 5 aborted fetuses. Briefly, samples were homogenized in phosphate-buffered saline (10% wt/vol) and prepped with 0.4-µM selective filtration and nuclease treatment, and DNA was extracted as previously described (2, 3). Sequencing libraries were prepared using the NexteraXT kit (Illumina) according to the manufacturer’s instructions and quantified with the Kapa library quantification kit for Illumina platforms (Kapa Biosystems), and fragment length distribution was verified using the Bioanalyzer with the High Sensitivity DNA kit (Agilent Technologies). Sequencing was performed on a MiSeq sequencer using a MiSeq reagent kit version 3 (Illumina) with 2 × 300-bp paired-end sequencing. Eighteen libraries were multiplexed using standard Illumina indexing primers.

The quality of the sequences was checked with the FastQC tool version 0.10.1 (http://www.bioinformatics.babraham.ac.uk/projects/fastqc). Stretches containing unidentified nucleotides (“N”) were trimmed using Cutadapt version 1.3 (4) prior to quality-trimming using Sickle version 1.210 (*Q* score <30, length <50 bp) (5).

For the metagenomics analysis, rRNA reads and host reads were removed using the SortMeRNA tool version 1.7 (6) and mapping against the bovine reference genome (GenBank accession nos. AC_000158.1 to AC_000187.1, NC_006853.1) using BWA-MEM version 0.7.5a-r405 (7). Remaining reads were subjected to a megablast sequence alignment analysis against the NCBI nucleotide database using blast-2/2/27+ software (8), with an *E* value threshold of 0.001, followed by taxonomy assignment using MN version 4.70.4 (9, 10). Bovine polyomavirus type 1 (BPyV-1) sequences were present in all samples and could be confirmed in 5 out of 18 tested samples using real-time RT-PCR (11).

Members of the family *Polyomaviridae* are small, nonenveloped, double-stranded DNA viruses, which are widely distributed among vertebrates (12). They share a genome structure consisting of a 4.8- to 5.5-kbp circular double-stranded DNA. A BPyV-1 is unlikely to be causally involved in the abortion cases, as it is a known persistent contaminant of cattle that has been detected in bovine sera (13, 14), meat products (15, 16), and even waste and surface water (17–19).

A total of 50,230 quality-trimmed sequences from a highly positive liver sample were mapped against a reference genome (GenBank accession no. KM496323) using GS Mapper version 2.9 (Roche), resulting in a 4,697-nucleotide contig corresponding to the complete genome length. The protein-coding genes were predicted relative to reference sequence NC_001442 by GATU (20) and supplemented with manual annotation for spliced genes.

The complete genome of this bovine polyomavirus type 1 (BPyV/BEL/1/2014) is characterized as a double-stranded circular DNA comprising 4,697 bp, with an average GC content of 41.41%, and containing 6 protein-coding genes.

### Nucleotide sequence accession number.

The complete genome sequence of BPyV/BEL/1/2014 was deposited in DDBJ/EMBL/GenBank under the accession number KU200259.

## References

[1] Delooz L, Mori M, Petitjean T, Evrard J, Czaplicki G, Saegerman C 2015 Congenital jaundice in bovine aborted foetuses: an emerging syndrome in southern Belgium. Transbound Emerg Dis 62:124–126. doi:10.1111/tbed.12326. 25620571

[2] Rosseel T, Pardon B, De Clercq K, Ozhelvaci O, Van Borm S 2014 False-positive results in metagenomic virus discovery: a strong case for follow-up diagnosis. Transbound Emerg Dis 61:293–299. doi:10.1111/tbed.12251. 24912559

[3] Rosseel T, Ozhelvaci O, Freimanis G, Van Borm S 2015 Evaluation of convenient pretreatment protocols for RNA virus metagenomics in serum and tissue samples. J Virol Methods 222:72–80. doi:10.1016/j.jviromet.2015.05.010. 26025457

[4] Martin M 2011 Cutadapt removes adapter sequences from high-throughput sequencing reads. EMBnet J 17:10–12. doi:10.14806/ej.17.1.200.

[5] Joshi NA, Fass J 2011 Sickle: A sliding-window, adaptive, quality-based trimming tool for FastQ files. Version 1.33 https://github.com/najoshi/sickle.

[6] Kopylova E, Noé L, Touzet H 2012 SortMeRNA: fast and accurate filtering of ribosomal RNAs in metatranscriptomic data. Bioinformatics 28:3211–3217. doi:10.1093/bioinformatics/bts611. 23071270

[7] Li H 2014 Toward better understanding of artifacts in variant calling from high-coverage samples. Bioinformatics 30:2843–2851. doi:10.1093/bioinformatics/btu356. 24974202 PMC4271055

[8] Tao T 2010 Standalone BLAST setup for Windows PC. In BLAST® help [Internet]. NCBI, Bethesda, MD http://www.ncbi.nlm.nih.gov/books/NBK52637.

[9] Huson DH, Auch AF, Qi J, Schuster SC 2007 MegaN analysis of metagenomic data. Genome Res 17:377–386. doi:10.1101/gr.5969107. 17255551 PMC1800929

[10] Huson DH, Mitra S 2012 Introduction to the analysis of environmental sequences: metagenomics with MEGAN. Methods Mol Biol 856:415–429. doi:10.1007/978-1-61779-585-5_17. 22399469

[11] Hundesa A, Bofill-Mas S, Maluquer de Motes C, Rodriguez-Manzano J, Bach A, Casas M, Girones R 2010 Development of a quantitative PCR assay for the quantitation of bovine polyomavirus as a microbial source-tracking tool. J Virol Methods 163:385–389. doi:10.1016/j.jviromet.2009.10.029. 19887085

[12] Pérez-Losada M, Christensen RG, McClellan DA, Adams BJ, Viscidi RP, Demma JC, Crandall KA 2006 Comparing phylogenetic codivergence between polyomaviruses and their hosts. J Virol 80:5663–5669. doi:10.1128/JVI.00056-06. 16731904 PMC1472594

[13] Van der Noordaa J, Sol CJ, Schuurman R 1999 Bovine polyomavirus, a frequent contaminant of calf sera. Dev Biol Stand 99:45–47. 10404875

[14] Wang J, Horner GW, O’Keefe JS 2005 Detection and molecular characterisation of bovine polyomavirus in bovine sera in New Zealand. NZ Vet J 53:26–30. 10.1080/00480169.2005.36465 15731831

[15] Peretti A, FitzGerald PC, Bliskovsky V, Buck CB, Pastrana DV 2015 Hamburger polyomaviruses. J Gen Virol 96:833–839. doi:10.1099/vir.0.000033. 25568187 PMC4361794

[16] Zhang W, Li L, Deng X, Kapusinszky B, Delwart E 2014 What is for dinner? Viral metagenomics of US store bought beef, pork, and chicken. Virology 468–470:303–310. doi:10.1016/j.virol.2014.08.025. PMC4252299 25217712

[17] Corsi SR, Borchardt MA, Spencer SK, Hughes PE, Baldwin AK 2014 Human and bovine viruses in the Milwaukee River watershed: hydrologically relevant representation and relations with environmental variables. Sci Total Environ 490:849–860. doi:10.1016/j.scitotenv.2014.05.072. 24908645 PMC7125695

[18] Lazic G, Grubac S, Lupulovic D, Bugarski D, Lazic S, Knezevic P, Petrovic T 2015 Presence of human and animal viruses in surface waters in Vojvodina province of Serbia. Food Environ Virol 7:149–158. doi:10.1007/s12560-015-9187-3. 25687987

[19] Wong K, Xagoraraki I 2011 Evaluating the prevalence and genetic diversity of adenovirus and polyomavirus in bovine waste for microbial source tracking. Appl Microbiol Biotechnol 90:1521–1526. doi:10.1007/s00253-011-3156-z. 21394527

[20] Tcherepanov V, Ehlers A, Upton C 2006 Genome annotation transfer utility (GATU): rapid annotation of viral genomes using a closely related reference genome. BMC Genomics 7:150. doi:10.1186/1471-2164-7-150. 16772042 PMC1534038

